# Social Cues Alter Implicit Motor Learning in a Serial Reaction Time Task

**DOI:** 10.3389/fnhum.2018.00197

**Published:** 2018-05-14

**Authors:** Alexander Geiger, Axel Cleeremans, Gary Bente, Kai Vogeley

**Affiliations:** ^1^Institute of Neuroscience and Medicine, Cognitive Neuroscience (INM-3), Research Centre Juelich, Juelich, Germany; ^2^Brain Imaging Lab, Department of Psychiatry, University Hospital Cologne, Cologne, Germany; ^3^Consciousness, Cognition & Computation Group, Center for Research in Cognition & Neurosciences, ULB Neuroscience Institute, Université Libre de Bruxelles, Bruxelles, Belgium; ^4^Department of Psychology, Faculty of Human Sciences, University of Cologne, Cologne, Germany; ^5^Department of Communication, Michigan State University, East Lansing, MI, United States

**Keywords:** social cognition, social interaction, nonverbal communication, social gaze, serial reaction time task, implicit motor learning, social/non-social cueing

## Abstract

Learning is a central ability for human development. Many skills we learn, such as language, are learned through observation or imitation in social contexts. Likewise, many skills are learned implicitly, that is, without an explicit intent to learn and without full awareness of the acquired knowledge. Here, we asked whether performance in a motor learning task is modulated by social vs. object cues of varying validity. To address this question, we asked participants to carry out a serial reaction time (SRT) task in which, on each trial, people have to respond as fast and as accurately as possible to the appearance of a stimulus at one of four possible locations. Unbeknownst to participants, the sequence of successive locations was sequentially structured, so that knowledge of the sequence facilitates anticipation of the next stimulus and hence faster motor responses. Crucially, each trial also contained a cue pointing to the next stimulus location. Participants could thus learn based on the cue, or on learning about the sequence of successive locations, or on a combination of both. Results show an interaction between cue type and cue validity for the motor responses: social cues (vs. object cues) led to faster responses in the low validity (LV) condition only. Concerning the extent to which learning was implicit, results show that in the cued blocks only, the highly valid social cue led to implicit learning. In the uncued blocks, participants showed no implicit learning in the highly valid social cue condition, but did in all other combinations of stimulus type and cueing validity. In conclusion, our results suggest that implicit learning is context-dependent and can be influenced by the cue type, e.g., social and object cues.

## Introduction

In everyday life, humans often share their inner experiences with others non-verbally, making use of gestures, facial expressions or gaze behavior. Nonverbal communication, which can be unintentionally conveyed by the sender, serves different functions, including coordination, discourse, dialog and socio-emotional functions (Sugawara et al., 2012).

A special channel through which we exchange information with others is social gaze behavior (Argyle and Cook, 1976). The human eyes, compared to the eyes of other primates, have a unique morphology, amongst which a white sclera that makes it possible to identify the gaze direction of conspecifics even from large distances (Kobayashi and Kohshima, 2001).

This morphology might have developed as a remote non-verbal communication signal. With a simple gaze-shift, we are able to redirect the attention of another person to a specific object in the environment (e.g., gazing at a potential food source or an approaching enemy), and thus enable joint attention (Baron-Cohen, 1997; Emery, 2000; Kampe et al., 2003; Frischen et al., 2007). The eyes of other people thus expand one’s personal visual domain through cognitive attribution. Eye contact, coupled with theory-of-mind-driven mechanisms, subtend non-verbal social communication in humans and animals (Butterworth and Cochran, 1980; Baron-Cohen, 1992; Emery, 2000) and shape the initiation and regulation of social interaction (Argyle and Cook, 1976; Macrae et al., 2002; Kuzmanovic et al., 2009). The interpretation of the gaze-behavior of another person, despite its inherent ambiguity, is thus a very salient cue in social interaction and can convey multi-faceted, rich information about both the other persons state of mind as well as about important features of the environment in which both agents are interacting.

Many studies dedicated to studying gaze-behavior have used virtual characters (avatars or agents), which make it possible to have full control of the facial expressions and eye movements so as to provoke similar reactions in the perceivers as real human agents (Bente et al., 2001; Bailenson et al., 2003; Slater et al., 2006; Sugawara et al., 2012; Georgescu et al., 2014).

An interesting experimental design to investigate the relative influence of different stimuli (e.g., social and object stimuli) on behavior and on awareness are cueing paradigms (Posner, 1980). Beyond the traditional object cues (i.e., arrows) used in typical Posner paradigms, different studies have now investigated the influence of gaze-shifts. Adults (Friesen and Kingstone, 1998; Driver et al., 1999; Hietanen, 1999) and even 4–5 month-old infants (Hood et al., 1998) reacted with gaze-following to the perceived gaze-shifts of others. Congruently, using a Stimulus-Response-Compatibility- (SRC) task, we showed that gaze influences action control, resulting in increased accuracy and faster reaction times (RTs; Schilbach et al., 2011, 2012). The effect of social cues on motor control is also corroborated by findings indicating that social cues activate a different neural network compared to object cues (Kingstone et al., 2004; Hietanen et al., 2006; Tipper et al., 2008; Greene et al., 2009).

In this article, we investigate to what extent a social context, or in this case gaze information, can modulate high-level motor actions. To address this question, we used a dual-stimulus experiment that engages implicit motor learning and compared the influence of different cues (i.e., a social cue or a object cue) on motor performance.

Most of everyday actions need to be conducted in a distinct sequence of specific movements. Lashley (1951) postulated that sequences of specific actions play a fundamental role in movements and behavior. For example preparing a meal consists of specific actions, like taking a pan, cutting the vegetables and putting them into the pan.

Humans have to be sensitive to sequences or regularities in their actions and recognize dependencies to create the correct sequence of movements. Interestingly, it is still unclear how humans are able to learn these correct sequences of events. According to one interpretation, the acquisition of the knowledge of such sequences is facilitated to the knowledge of explicit rules and extended practice (e.g., Anderson, 1983, 1987). After practice, humans do not need the knowledge about what body movement is needed, for example to ride a bike.

Many skills that require a distinct sequence of actions seem to be acquired without the knowledge of explicit rules and hidden structures. This kind of acquiring new knowledge without conscious awareness of the learning process is described as implicit learning (e.g., Reber, 1967), or for the example of actions, implicit motor learning.

A typical paradigm in the domain of implicit motor learning is the “Serial Reaction Time (SRT)”-Task (SRT-Task, see Nissen and Bullemer, 1987). In such sequence learning situations, people are simply asked to respond as fast and as accurately as possible to the appearance of a stimulus at one of several locations (typically 4–6) on a computer screen by pressing on a spatially corresponding key. The instructions typically emphasize both speed and precision but highlight the need to produce speeded responses. Unbeknownst to participants, the stimulus does not move about randomly, but rather follows a repetitive sequence of successive locations in the majority of the blocks. With practice, participants become progressively sensitive to the sequential structure of the material, which enables them to anticipate the next location at which the stimulus will appear and hence to reduce their response times (RTs). Learning is typically objectively measured by assessing the extent to which the RT gains observed with a trained sequence turn into interference when a novel, unfamiliar sequence is introduced (Reed and Johnson, 1994; Destrebecqz and Cleeremans, 2001; Coomans et al., 2014). A striking feature of such paradigms is that most participants tend to remain subjectively unaware of the fact that the material contains sequential structure, and often experience difficulty leveraging the acquired knowledge in direct tests of awareness, such as in recognition (i.e., “is this sequence fragment part of the training sequence”) or generation (i.e., “please produce a sequence that resembles the training sequence”) tasks. These observations have led many to conclude that sequence learning is an instance of implicit learning, though there is continuing debate about the extent to which knowledge acquired in such paradigms is indeed fully unconscious (Shanks and John, 1994). A fair conclusion from over two decades of research on sequence learning is that people’s ability to verbalize the acquired knowledge systematically lags behind their ability to deploy it in the training context (Willingham et al., 1989). While we did assess awareness in our experiment, it is important to note that this was not the main focus of the study, which was instead dedicated to exploring the relative influences of social vs. object cues on performance. To address this issue, we designed a dual-stimulus experiment inspired by the work of Cleeremans (1995). Cleeremans investigated the influence of pointing arrows, i.e., object cues, with different cueing validities on implicit motor learning in a dual-stimulus experiment. In this type of preparation, two potentially competing sources of information can thus be used to prepare the next motor response: (Implicit) Information conveyed by the extent to which a stimulus is predictable based on the temporal context set by previous elements of the sequence, and (explicit) information conveyed by the cue, which may or may not correctly indicate where the next stimulus will appear. The latter can be varied parametrically to modulate the validity of the cue and presumably, people’s reliance on it when preparing the next response.

In our study, we adapted this design so as to contrast social vs. object cues, either by using a cartoon human face composed of geometric figures for the social condition, or by re-arranging the figures in such a way that no face or other human “gestalt” was present in the object condition. In addition, we wanted to examine whether different cue-types and cueing validities could alter participants’ awareness of the sequential pattern of the target stimulus, and thus the learning of complex relationships between the stimuli. Based on the assumption that implicit learning is an automatic process, we expected implicit motor learning to take place even in conditions that involve highly valid cueing, as Cleeremans (1995) demonstrated.

The overall design of the experiment is depicted in Table 1. It consisted of a 2 × 2 between-subject design with CUE (social (“SOC”) vs. object (“OBJ”)) and VALIDITY (high validity (“HV”) vs. low validity (“LV”)) as independent factors. In the following, we refer to the different conditions using as follows: SOC-HV (social cue—high validity), SOC-LV (social cue—low validity), OBJ-HV (object cue—high validity), OBJ-LV (object cue—low validity).

**Table 1:** Blocks with S/s were blocks with the training sequence (Seq_1), R represents the first transfer block with another, unknown sequence (Seq_2), r represents the second transfer block with the second unknown sequence (Seq_3).

1	2	3	4	5	6	7	8	9	10	11	12	13
S	S	S	S	S	S	S	S	R	S	s	r	s

*In blocks with capital letters, additional directional cues were given by either the social or object stimulus. In blocks with lower case letters, no additional directional cues were given*.

We propose the following hypothesizes:

1. High-validity cues (vs. low-validity cues) lead to faster responses independently of the stimulus type (social vs. object).
2. Social cues (vs. object cues) lead to faster responses independently of cue validity (in reference to Schilbach et al., 2011, 2012).
3. The combinations of stimulus type and cueing validity have differential effects on motor responses. Based on the findings of Schilbach et al. (2011, 2012), we assume that in the low validity condition, the social cue will lead to faster responses than the object cue.
4. Concerning implicit motor learning, we hypothesized that the presence of an explicit cue would overwrite (in the high validity condition), or interfere (in the low validity condition) with implicit learning, independent of the stimulus type.

## Materials and Methods

### Participants

Sixty persons provided written informed consent and participated in the study. This study was carried out in accordance with the recommendations of the local ethics committee of the University Hospital Cologne with written informed consent from all subjects. All subjects gave written informed consent in accordance with the Declaration of Helsinki. The protocol was approved by the the local ethics committee of the University Hospital Cologne. The participants were randomly assigned to one of the four conditions. Three participants were excluded from the analysis because their RTs deviated by more than 2 SD. The final assignment of participants and conditions was: SOC-HV: 15 participants (seven male), age: 27.53 ± 4.37; SOC-LV, 14 participants (six male), age: 24.50 ± 3.88; OBJ-HV: 14 participants (six male), age: 23.64 ± 3.54; OBJ-LV, 14 participants (six male), age: 25.11 ± 3.66.

All participants were right-handed according to the Edinburgh handedness inventory (EHI; Oldfield, 1971), had no history of psychiatric or neurological disease and were not taking any neuro- or psychotropic medication. All remaining volunteers had a normal or corrected to normal vision and were naïve to the purpose of the study. Besides the EHI, the following measures were obtained: IQ was assessed by a German multiple choice vocabulary test (“Wortschatztest”, WST; Schmidt and Metzler, 1992), Autism Quotient (Baron-Cohen et al., 2001), Empathy Quotient (Baron-Cohen and Wheelwright, 2004), Systemizing Quotient (Baron-Cohen et al., 2003), Beck Depression Inventory (Beck et al., 1961) to rule out clinically relevant symptoms of depression.

### Apparatus and Display

Subjects were seated in front of an LCD monitor at a distance of 72 cm, which was kept constant by using an adjustable chin-rest. The gaze-behavior of the participants, i.e., the fixation of a fixation cross in the middle of the screen, was recorded via an eye-tracking device (EyeLink1000, 1 kHz, SR Research Ltd. Kanata, ON, Canada). RTs were recorded by the Presentation^®^ software (Version 0.70^1^).

## Materials and Procedure

### Dual-Stimulus SRT-Task

We used a modified SRT-Task (Nissen and Bullemer, 1987). The target stimulus, a black X, was presented at one of four locations marked by four gray squares arranged along a horizontal line on the screen. Each position corresponded to one of four keys ([v], [b], [n], [m]) on a German QWERTZ keyboard. The spatial configuration of the keys was fully compatible with the position of the squares on the screen. A fixation cross was located 3 cm above the middle of the four boxes; participants were asked to fixate it throughout the experiment. Subjects were asked to press the key corresponding to the current location of the stimulus as fast and accurately as possible. Stimuli remained visible until the subjects responded with a button press.

The experiment consisted of 13 blocks with 84 trials each, resulting in 1092 trials in total. The sequences consisted of 12 locations and were repeated seven times per block, starting at a randomly selected position so as to impede explicit learning of the sequences. The sequences themselves were second-order conditional sequences (Reed and Johnson, 1994), characterized by the fact that perfect prediction of each sequence element depended on the identity and order of the two previous elements. Further, each location was as likely as any other to follow any of the other three locations. In addition, each location appeared equally often, and no location was repeated back-to-back.

During blocks 1–8, 10, 11 and 13, one particular training sequence was presented to participants (Seq_1: 3-4-1-4-3-2-4-2-3-1-2-1; with “1” representing the far left location of the stimulus on the screen, and “4” representing the far right location).

In two “transfer blocks” (blocks 9 and 12), two different sequences based on the same building principles as described above were presented, namely in block 9 (Seq_2: 3-4-1-2-4-3-1-4-2-1-3-2) and block 12 (Seq_3: 3-4-2-1-3-1-4-1-2-3-2-4).

In these “transfer blocks”, RT typically increases. This interference effect reflects participants’ sensitivity to the sequential structure of the main training sequence (Seq_1). Hence, the RT difference between training and transfer blocks can be used to compute a sequence-specific learning score (SLS). Sequences with similar difficulty were used to prevent the confounding of the sequence-specific learning effect due to differences in sequence structure as it might occur with pseudo-) random sequences (Reed and Johnson, 1994; Hoffmann and Koch, 1998). Between blocks, the German word “PAUSE” (Eng. “break”) indicated a break after which the participant her/himself could continue voluntarily via button press.

### Cues

**Figure 1:**
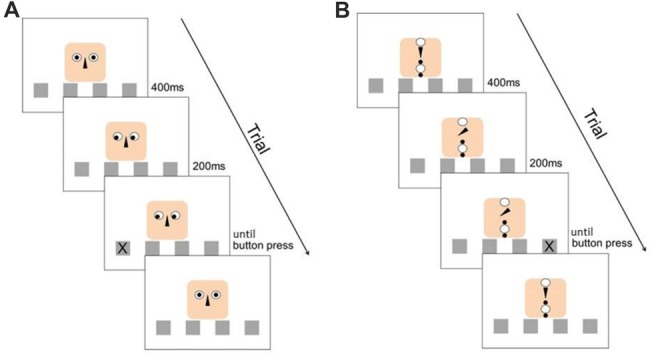
Experimental design. **(A)** Structure of a valid trial with the social stimulus. **(B)** Structure of an invalid trial with the object stimulus. The distribution of valid and invalid cues was balanced through the experiment for both stimulus types.

This classical SRT-task design was enriched by a second stimulus (the cue), which could either be “social” or “object” (see Figure 1). In the social context, the cue consisted of a cartoon human face composed of a finite number of simple geometric figures. The eyes of the face-like shape could gaze at one of the four locations and therefore provide an explicit directional cue about the location at which the next stimulus would appear. In the object context, the elements were re-arranged in such a way that they did not form any coherent “gestalt”-like figure, especially no face. Here, an arrow (representing the nose in the facial arrangement) was used as cueing device, pointing at the different locations. With this setup, we ensured that the perceived visual information was the same for both stimuli, and all measured differences in RTs were dependent on the perception of a face as opposed to an abstract arrangement of geometric figures as cueing signal. Irrespective of the stimulus type (social, or object), the target stimulus “X” appeared in one of the four boxes 200 ms after the directional cueing (see Figure 1).

Both social and object cues could vary in their validity. In the HV condition, the cue was always pointing at the location where the target stimulus would appear (“valid” cues) next. In the LV condition, the cue indicated the correct location in only half of the trials (“valid” cues) whereas in the other half of the trials the cue was randomly pointing at one of the three other locations (“invalid” cues). These invalid trials were irregularly distributed throughout the 12 digits of the sequence. The cues were present throughout blocks 1–10 (cued blocks; capital letters). In the last three blocks (11–13), the cue was still present, but “silent” in the sense that it did not move and thus did not provide any directional information (absence of either gaze-shift or arrow movement; uncued blocks, lower case letters). With this design, we aimed at investigating the influence of cue type and cueing validity on: (1) motor performance in general; (2) the extent to which learning was implicit; and (3) whether participants were able to recall the implicitly learned sequence in the absence of additional directional cues.

To avoid any carry-over effects of cue type or validity, each participant only performed one of the four conditions.

After the SRT task, explicit motor sequence knowledge was assessed by using a standardized structured interview and a free sequence generation task. First, participants were asked if they had noticed anything over the course of the experiment that they had not been told before. Second, if participants failed to report having noticed anything particular, they were asked if they had identified any regularities in the succession of the stimuli. If participants did not mention anything concrete, they were then asked whether they thought that the sequence had been random. Participants who still described the sequence as being random were then told about the underlying structure of the material.

### Generation Task

After the SRT task and the interview, all participants underwent a generation task (see Destrebecqz and Cleeremans, 2001). Here, to assess the extent to which the acquired knowledge was available to awareness, we adapted Jacoby’s (1991) process dissociation procedure to disentangle implicit and explicit knowledge of the hidden sequence in the SRT task. Participants performed a “free generation” task, previously shown to be a sensitive test of sequence knowledge (Perruchet and Amorim, 1992), under either “inclusion” or “exclusion” generation instructions.

The generation task resembled the structure of the experiment: four location squares were presented, but neither cues not stimuli were presented. Participants were told that every target location was equally distributed within the 12 digit training sequence and that no target location was presented two times in a row. Under inclusion instructions, participants were then asked to freely generate a series of 24 button presses that “resembled the training sequence as much as possible.” Next, participants were asked to generate another series of 24 button presses trials under exclusion instructions, that is, to try to avoid producing the pattern of the training sequence. We assumed, following Destrebecqz and Cleeremans (2001), that the observation that participants are unable to refrain from producing familiar fragments of the training sequence under exclusion instructions is reflective of implicit learning, for it suggests that participants are unable to recognize the relevant fragments and inhibit their production. To obtain an index of implicit learning, we computed the number of familiar triplets (triplets are the correct length with SOC sequences, see (Destrebecqz and Cleeremans, 2001), for discussion), produced under both inclusion and exclusion instructions.

### Statistical Analyses

Statistical analyses were performed with the statistical software package SPSS (Version21, IBM) and R (Version 3.1.0). For the analyses, only blocks 2–13 were used. Block 1 was considered to be a training block and was therefore excluded from further analysis.

The first trial of all remaining blocks was also rejected. Furthermore, all incorrect trials (trials with a spatially non-congruent button-press) and trials following such incorrect responses were also discarded from the data set so to reduce the noise resulting from post error slowing (Rabbitt, 1966), which occurs independently of sequence learning. In addition, all responses faster than 100 ms or slower than 1000 ms were also excluded from further analysis. The median RT per block was then calculated for each individual subject. This value was then used to compute mean RTs for each block on group level.

For the analysis of variance, effects of BLOCKS (2–8) were used as within-subject factor. CUE and VALIDITY were used as between-subject-factors. If Mauchly’s test indicated a violation of the assumption of sphericity, the Greenhouse-Geisser correction was used.

A SLS was calculated by comparing the RTs of block 9 (the transfer block) with the average of the RTs obtained in the adjacent blocks (blocks 8 and 10) for the cued condition. SLS was also calculated for the uncued condition (blocks 11, 13 and block 12 as transfer block; see Dovern et al., 2011; Wierzchón et al., 2012). These SLSs reflect the differences in RT for the trained and untrained sequence and thus reflect the amount of implicit knowledge of the training sequence with the presence or absence of directional cues.

In addition, the incongruency costs in the LV condition were calculated, this value reflects the extra “computational load” associated with the inhibition of the reflexive congruent response, processes of attentional reorienting and the generation of a incongruent motor response (Proctor and Reeve, 1989; Matsumoto et al., 2004; Greene et al., 2009; Schilbach et al., 2011, 2012).

The generation score for the inclusion and exclusion generation part was computed by creating all possible triplets of pushed buttons (22 triplets) and comparing the number of correct triplets within the double number of triplets of the trained sequence (24 triplets of the training sequence).

## Results

### Error Rates

The analysis of the amount of incorrect responses for blocks 2–8 showed that all participants exhibited a mean error rate smaller than 5% in all four conditions. Error rates provided no suitable measure for the learning effect and thus were not further analyzed. These results show that participants were able to follow the given instructions and managed not to be substantially distracted by the invalid cues in the LV condition.

### Reaction Times

**Figure 2:**
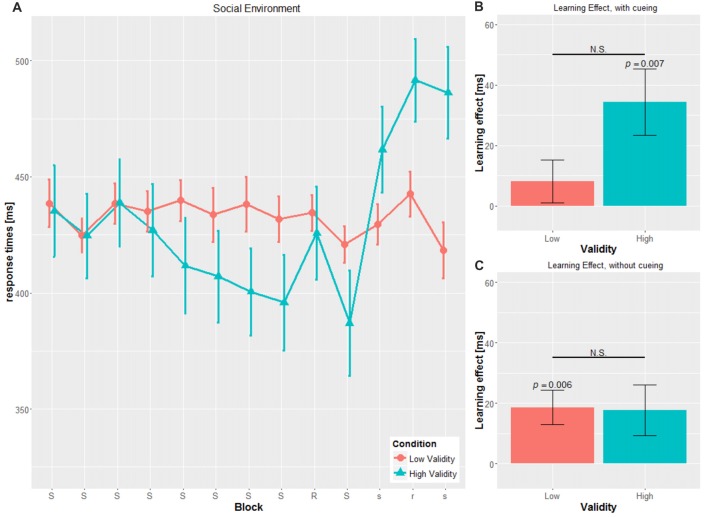
Main results for the social environment. **(A)** RT and SE for the two validity conditions throughout the experiment **(B)** learning effect for cued blocks (S-R-S) with SE **(C)** learning effect for uncued blocks (s-r-s) with SE; RT, reaction time; SE, Standard error, N.S., non-significant.

**Figure 3:**
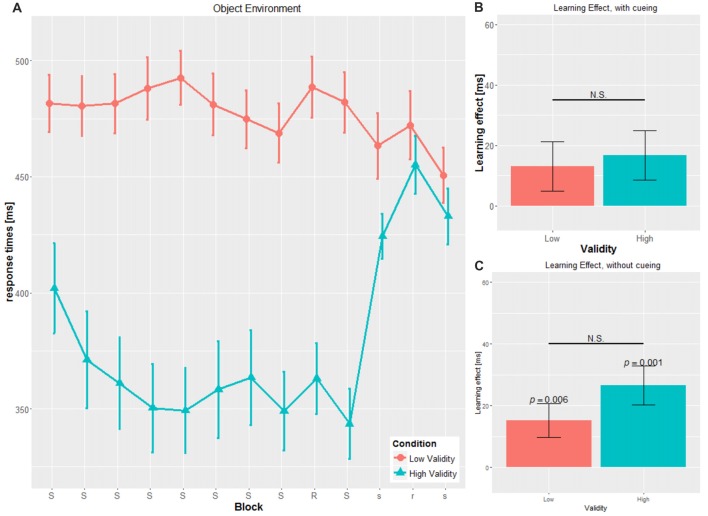
Main results for the object environment.** (A)** RT and SE for the two validity conditions throughout the experiment **(B)** learning effect for cued blocks (S-R-S) with SE **(C)** learning effect for uncued blocks (s-r-s) with SE; RT, reaction time; SE, Standard error, N.S., non-significant.

A mixed-design ANOVA was conducted with CUE and VALIDITY as between-subject factor and BLOCK, i.e., blocks with the cueing stimulus (block 2 to block 8), as within-subject variables. For the dependent variable, mean RT on group-level was used (see Figures 2A, 3A).

**Confirming the first hypothesis**, the analysis revealed a significant main effect of VALIDITY (*F*_(1,53)_ = 23.326, *p* < 0.001, *η*^2^ = 0.306), with significantly faster RTs in the HV condition compared to the LV condition, irrespective of cue type (HV: 387.28 ± 13.73 ms, LV: 457.83 ± 43.75 ms, *t*_(55)_ = 4.365, *p* < 0.001).

No significant main effect for CUE (*F*_(1,53)_ = 0.138, *p* = 0.711, *η*^2^ = 0.003) was found. Cue type by itself seems not to have an impact on RTs, which is in contrast to our **second hypothesis**. However, confirming the **third hypothesis**, we did find a significant interaction for CUE × VALIDITY (*F*_(1,49)_ = 12.331, *p* = 0.001, *η*^2^ = 0.189), with significant differences in the object conditions only (OBJ-HV: 357.53 ± 66.85 ms; OBJ-LV: 481.08 ± 43.01; *t*_(22.19)_ = 5.816, *p* < 0.001), but not in the social conditions (SOC-HV: 415.05 ± 71.18 ms; SOC-LV: 434.58 ± 3100 ms; *t*_(19.404)_ = 0.969, *p* = 0.345).

By comparing the RTs within the HV condition for the two cue types, the object cue elicits significantly faster RTs throughout blocks 2–8 compared to the social cue (*t*_(27)_ = 2.239, *p* = 0.034).

The LV condition led to significantly different RTs for the two cue types, with faster RTs for the SOC-LV (SOC-LV: 434.58 ± 31.00 ms, OBJ-LV: 481.08 ± 43.01; *t*_(26)_ = 3.282, *p* = 0.003).

Concerning the temporal aspects of the experiment, we found a significant main effect for BLOCK (*F*_(3.884, 205.860)_ = 3.043, *p* = 0.019, *η*^2^ = 0.054), with an RT decrease within the training blocks 2–8, meaning that the participants responded faster throughout the experiment, independently of cue type or cue validity, either due to the familiarity of the task, or to implicit learning of the hidden sequence.

Neither the BLOCK × CUE (*F*_(3.884, 205.860)_ = 1.008, *p* = 0.403, *η*^2^ = 0.019) interaction nor the three-way BLOCK × CUE × VALIDITY (*F*_(3.884, 205.860)_ = 2.260, *p* = 0.066, *η*^2^ = 0.041) interaction achieved significance. The interaction BLOCK × VALIDITY (*F*_(3.884, 205.860)_ = 2.440, *p =* 0.05, *η*^2^ = 0.044) showed a tendency for faster RTs in later blocks in the HV-condition. In the LV-condition, RTs did not increase over time, which would be in line with the third hypothesis, saying that low valid cueing disrupts implicit learning of the hidden sequence.

To investigate how motor responses changed in the absence of any directional cues, the mean for the last three cued blocks (blocks 8, 9, 10) for each condition for each participant was calculated and compared to the mean of the uncued blocks (blocks 11, 12, 13). *T*-tests showed that only in the SOC-HV condition RTs were significantly slower in the absence of cueing (mean cued blocks: 402.87 ± 77.00 ms, mean uncued blocks: 479.84 ± 69.93 ms, *t*_(14)_ = 6.578, *p* < 0.001), but not in the SOC-LV condition (mean cued blocks: 429.08 ± 26.25 ms, mean uncued blocks: 430.19 ± 33.75 ms, *t*_(13)_ = 0.250, *p* = 0.806).

In the uncued blocks, SOC-HV and SOC-LV do significantly differ in their mean RT (*t*_(20.485)_ = 2.460, *p* = 0.023).

In the object cue condition, we found a similar pattern for OBJ-HV with slower RT in the absence of cueing (mean cued blocks: 351.90 ± 55.70 ms, mean uncued blocks: 437.48 ± 40.289 ms, *t*_(13)_ = 8.586, *p* < 0.001), but the opposite pattern in the OBJ-LV condition with faster RT in the absence of cueing (mean cued blocks: 479.82 ± 41.66 ms, mean uncued blocks: 462.05 ± 48.79 ms, *t*_(13)_ = 2.588, *p* = 0.022).

In the uncued blocks, OBJ-HV and OBJ-LV did not differ in their mean RTs (*t*_(26)_ = 1.453, *p* = 0.158).

Taken together, our results confirm that VALIDITY did alter motor actions of the participants with faster RTs during HV cueing compared to LV cueing, independently of cue type, as proposed in hypothesis one.

The two cue types had different impacts on the motor responses depending on cueing validity. When highly valid, the object cue led to faster responses than the social cue. In contrast, when the validity of the cue was low, the social cue led to significantly faster responses compared to the object cue.

In the HV condition, the uncued blocks led to significant slower RTs compared to the cued blocks, irrespective of the cue. In addition, the uncued blocks in the SOC-HV and SOC-LV differed significantly, but not between the OBJ-HV and OBJ-LV conditions. In the SOC-LV condition, RTs were not significantly different in the uncued blocks compared to the cued blocks. This is in contrast to the OBJ-LV condition, in which the mean of the uncued blocks was significantly faster than the mean RT of the cued blocks. These findings are in line with third hypothesis. Interestingly, only the combination of stimulus type and cueing validity but not the stimulus itself has an impact on the given responses, which is in contrast to hypothesis two.

### Sequence-Specific Learning Score—Cued

The SLS was defined as the difference of the transfer block (block 9) and the mean of the adjacent blocks (block 8 and block 10; see Figures 2B, 3B).

For the cued blocks, *t*-tests were performed to investigate if participants showed any implicit learning in the cueing blocks with mean values different from zero.

We found significant SLS in the cued blocks only for the SOC-HV (34.40 ± 42.629 ms, *t*_(14)_ = 3.125, *p* = 0.007), but not any of the other conditions (SOC-LV: 8.071 ± 26.419 ms, *t*_(13)_ = 1.143, *p* = 0.274; OBJ-HV: 16.804 ± 30.568 ms, *t*_(13)_ = 2.057, *p* = 0.06; OBJ-LV: 13.071 ± 30.371 ms, *t*_(13)_ = 1.610, *p* = 0.131).

Comparisons of the SLS for the cued block revealed a tendency to significance between SOC-HV and SOC-LV (*t*_(27)_ = 1.982, *p* = 0.058). This was not true for OBJ-HV and OBJ-LV (*t*_(26)_ = 0.324, *p* = 0.748). Concerning the different cueing validities, we did not find a significant difference neither between SOC-HV and OBJ-HV (*t*_(27)_ = 1.269, *p* = 0.215) nor between SOC-LV and OBJ-LV (*t*_(26)_ = 0.465, *p* = 0.646). This result suggests that participants were sensitive to the training sequence only in the SOC-HV condition, whereas in all other conditions participants seemed not to have implicitly learnt the hidden sequence.

### Sequence-Specific Learning Score—Uncued

We conducted the same computation for the SLS for the uncued blocks as for the SLS for the cued blocks (see Figures 2C, 3C). Results did not show a significant difference from zero for SOC-HV (17.683 ± 32.504 ms, *t*_(14)_ = 2.107, *p* = 0.054). However, significant difference from zero could be found for SOC-LV (18.625 ± 21.433 ms, *t*_(13)_ = 3.251, *p* = 0.006), OBJ-HV (26.554 ± 23.520 ms, *t*_(13)_ = 4.224, *p* = 0.001) and OBJ-LV (15.196 ± 20.450 ms, *t*_(13)_ = 2.780, *p* = 0.016).

Comparisons of the SLS for the uncued blocks reveal no significant differences between SOC-HV and SOC-LV (*t*_(27)_ = 0.091, *p* = 0.928), OBJ-HV and OBJ-LV (*t*_(26)_ = 1.363, *p* = 0.184), for both HV conditions (*t*_(27)_ = 0.837, *p* = 0.410) or both LV conditions (*t*_(26)_ = 0.433, *p* = 0.669).

This finding suggests that as soon as the cue stopped giving directional indications, participants only showed implicit learning in the SOC-LV, OBJ-HV and OBJ-LV conditions. All other comparisons between the different cueing validities did not show any significant differences.

### Reaction Times for the LV, Divided in Valid and Invalid Cued Trials

To further investigate the LV-condition, we investigated the differences for the valid and invalid cued trials of the blocks 2–8. We used BLOCK and CUEING (i.e., valid and invalid cued trials) as within-subject and CUE as between-subject factor. The ANOVA revealed a significant main effect for CUEING (*F*_(1,26)_ = 57.074, *p* < 0.001, *η*^2^ = 0.687), due to faster reactions in valid cued trials compared to invalid cued trials (valid: 441.691 ± 47.158 ms, invalid: 471.939 ± 44.296 ms, *t*_(27)_ = 7.641, *p* < 0.001) independent of the cue and CUE (*F*_(1,26)_ = 10.391, *p* = 0.003, *η*^2^ = 0.286), due to faster responses when interacting with the social compared to the object cue (social: 447.316 ± 30.032 ms, object: 496.561 ± 43.215 ms, *t*_(26)_ = 3.501, *p* = 0.002). The main effect for BLOCK was not significant (*F*_(3.979,156)_ = 1.549, *p* = 0.194, *η*^2^ = 0.056). The interactions CUEING × CUE (*F*_(1,156)_ = 0.392, *p* = 0.537, *η*^2^ = 0.015), BLOCK × CUEING (*F*_(6,156)_ = 1.628, *p* = 0.143, *η*^2^ = 0.059), BLOCK × CUE (*F*_(6,156)_ = 0.897, *p* = 0.499, *η*^2^ = 0.033) and the three-way interaction BLOCK × CUEING × CUE (*F*_(6,156)_ = 1.221, *p* = 0.298, *η*^2^ = 0.045) did not yield a significant result.

We also compared the valid trials for the social and object stimulus and found a significant difference (*t*_(26)_ = 2.772, *p* = 0.01) due to faster responses when interacting with the social cue. This was also true when we analyze the incongruent trials (*t*_(26)_ = 3.501, *p* = 0.002).

### Generation Task

For the generation task, we computed the number of generated chunks of three elements that were part of the training sequence in both inclusion and exclusion tasks. Participants were asked to perform 24 trials; thus the maximum number of correct chunks was 22. To obtain inclusion and exclusion scores for each subject, we therefore divided the corresponding number of correct chunks by 22.

First, we performed a *t*-test to compare the inclusion and exclusion scores (inclusion score: 0.154 ± 0.014, exclusion score: 0.1385 ± 0.012, *t*_(53)_ = 0.979, *p* = 0.332). No significant difference was found.

We conducted a univariate ANOVA with the generation scores of the inclusion and exclusion part as dependent and VALIDITY and STIMULUS as independent variable.

For the “inclusion task”, no significant main effect for CUE (*F*_(1,50)_ = 0.4, *p* = 0.53, *η*^2^ = 0.008), VALIDITY (*F*_(1,50)_ = 0.128, *p* = 0.722, *η*^2^ = 0.003) and no significant interaction for CUE × VALIDITY (*F*_(1,50)_ = 0.016, *p* = 1.624, *η*^2^ = 0.208) was found.

The univariate ANOVA for the “exclusion generation” task did not yield any significant main effect for VALIDITY (*F*_(1,50)_ = 2.08, *p* = 0.651, *η*^2^ = 0.004), CUE (*F*_(1,50)_ = 0.004, *p* = 0.951, *η*^2^ = 0.0), nor the interaction CUE × VALIDITY (*F*_(1,50)_ = 0.345, *p* = 0.560, *η*^2^ = 0.0073).

These results suggest that the participants were not able to directly access any explicit knowledge of the training sequence.

## Discussion

The purpose of this study was to investigate the influence of different types of cues, i.e., social (SOC) and a object (OBJ), with two cueing validities, i.e., LV and HV, on a motor task. In addition, we investigated the effect of the interaction of cue type and cueing validity on the awareness of a hidden sequence in an implicit motor learning task. To our knowledge, this is the first study examining the effect of social and object stimuli with different cueing validity on motor processes in a dual-stimulus paradigm.

### Hypothesis 1: High Validity Cueing Leads to Faster Responses Than Low Validity Cueing

In line with the first hypothesis, our findings show that cueing validity has a substantial effect on motor performance: HV cueing lead to faster responses compared to the LV cueing in general, independent of the presented stimulus type.

This finding indicates that participants are using the directional valid cues to anticipate the location at which the next target stimulus will appear and are distracted by invalid cueing. This effect of cueing validity is well known and is in line with different other studies (Posner, 1980; Schilbach et al., 2011, 2012).

### Hypothesis 2: Social Cues Lead to Faster Responses Compared to the Object Stimulus

Concerning our second hypothesis, results showed no difference in RTs when looking at the cue type, i.e., social and object, in general. The cue type itself did not alter motor responses of the participants. Only the combination of the cue type and cueing validity led to significant differences in this experiment. When looking at the impact of cue type in the different cueing validities, we found a benefit of interacting with a social cue only in the LV condition, which is in accordance with Schilbach et al. (2011, 2012).

### Hypothesis 3: The Combinations of Stimulus Type and Cueing Validity Have Different Impacts on Motor Responses

While cueing validity shows a massive influence on RTs, cue type seems to have an effect on RTs only by its interaction with validity.

Within the HV condition, our findings show that cue type does interfere with the given responses due to the fact that RTs differ significantly between SOC-HV and OBJ-HV with the OBJ stimulus leading to faster responses. Interestingly, this finding is not in accordance with other studies. Previous findings suggest that social cueing stimuli lead to faster and more accurate responses compared to object stimuli (Macrae et al., 2002; Schilbach et al., 2011, 2012).

In the LV condition, the cue type also had a statistically significant impact on the motor performance, but here the social cue led to significantly faster responses compared to the object cue. The presented face might lead to higher alertness and thus lead to faster responses due to “social facilitation” (Zajonc, 1965; von Grünau and Anston, 1995; Farroni et al., 2002; Grossmann et al., 2007; Senju and Johnson, 2009). “Social facilitation” describes a performance-enhancing effect possibly based on increased physiological arousal, induced by the presence of another person (Quadflieg et al., 2004). The mere presence of another person, or only a face, lead to more pronounced motor, as well as inhibitory processes. In addition, this modulatory effect seems to help to coordinate one’s actions with those of another person (Schilbach et al., 2011, 2012). This described effect of “social facilitation” can also be found in the investigation of the valid and invalid trials in the LV condition. The social cue leads to significant faster responses compared to the corresponding object condition, irrespective of valid and invalid trials. In conclusion, our findings in the LV condition are consistent with previous results showing that social cues, i.e., a gaze-mediated social context, have a modulatory effect on action control (Kuzmanovic et al., 2009; Schilbach et al., 2011, 2012; Pfeiffer et al., 2013).

However, it is still unclear why participants react faster when interacting with the object cue in the HV condition in contrast to the social stimulus. One possible interpretation could be that participants do not attribute an inner motivation to the object stimulus and thus perceive it as a mere functional cue. In contrast, participants might assume a meaning behind the action of the social cue. This thinking about possible motivations might lead to slower responses when interacting with the social cue.

With respect to the mean of the cued and uncued blocks, participants’ performance was better with the assistance of the HV directional cues during the experiments. However, as soon as directional cues were absent, the RTs of the cued blocks got significantly slower in the uncued blocks and resembled the RTs of the LV condition when interacting with the object cue.

This finding is in line with a study of Pew (1974), showing that the benefit of different assistance methods did improve motor actions while being present but any benefits vanished, as soon as the participant had to perform a movement without the assistance.

When looking at the distracting cues, we found a different pattern of RTs for the cued and uncued blocks. Results showed no significant differences between the cued and uncued blocks in the SOC-LV condition but in the OBJ-LV condition, suggesting that the two cue types have different degrees of sustainability on motor actions. One possible explanation could be that participants are surprised to not get any directional cues again and try to understand why the face stopped moving. These mentalizing processes might overlay other motor processes and thus results do not show a decrease of RTs. These mentalizing processes might be absent when interacting with the object cue. Therefore, participants show faster RTs as soon as no distracting cueing stimuli are present.

### Hypothesis 4: Impact of Stimulus Type and Cueing Validity on Implicit Motor Learning

The results for the implicit sequential learning score (SLS) for the cued blocks show that participants seem to have learned the given sequence only in the SOC-HV condition, but not in any of the other conditions. The finding for the LV condition is in line with our hypothesis 4, saying the LV condition interferes with implicit learning and thus no learning effect could be observed. This effect could be explained by additional computational processes for inhibiting the inner motivation to follow the LV cues and thus this additional work might claim all attentional resources and thus reduce implicit motor learning processes.

Results showed a difference for the interaction of cue type and cueing: Only the SOC-HV condition led to implicit motor learning in the cued blocks, compared to the OBJ-HV.

First, these findings suggest that implicit motor learning is not an automatic process due to the fact that SLS greater than zero is only present in one of the four conditions, i.e., SOC-HV; the process of implicit motor learning seems to be dependent on the environment.

Second, results suggest that participants were able to take advantage of the predictability of the sequential pattern of the training sequence even when another, explicit source of information, i.e., the HV cueing of the social stimulus, is available. This effect was already described by Cleeremans (1995).

However, it is still unclear why participants do not show implicit learning when interacting with the OBJ-HV, but only with the SOC-HV condition in the cued blocks. One idea is that the OBJ-HV was perceived as less animate because no inner states of the person “behind the face” have to be taken into account and, hence, as more “trustworthy” compared to the SOC-HV, which would be in line with the analysis of the RTs.

In the OBJ-HV condition, participants appeared not to be sensitive and thus processed the hidden sequence. One possible explanation could be that the presence of a highly reliable source of information (the cue) results in a suppression of implicit learning processes that would normally be recruited during performance of the task. The cue is perceived as trustworthy and reliable so that implicit processing of the hidden sequential pattern is suppressed.

In contrast to the OBJ-HV, the SOC-HV condition showed an SLS in the cued blocks, meaning participants focused their attention on the task, and processed the hidden sequence implicitly without being influenced by the given directional cues. This finding suggests that the social face cue was perceived as less trustworthy and, hence, less reliable than the object cues, so that implicit learning processes were not suppressed or overwritten by provided cues.

Possibly participants intrinsically “think” that a person, i.e., the social cue, might accidentally make a mistake, even if it did not perform any mistakes throughout the whole experiment. These mentalizing processes might lead to a possible insecurity against the given cue and thus participants activate implicit learning processes.

Interestingly, no implicit learning was found in the SOC-HV condition in those blocks where no directional cues were given, meaning that participants were not able to recall the sequence when they had no assistance. This effect could also be found in Pew (1974). A possible explanation could be that participants were surprised that the social cue stopped giving cues and maybe start mentalizing why the eyes are not moving. These mentalizing processes might overlay any implicit motor learning processes.

Interestingly, we find a significant SLS in the OBJ-HV uncued blocks. Even if the pattern of RTs suggests that participants were not aware of the hidden sequence in the cued blocks, results show a learning effect in the uncued blocks. This leads to the assumption that participants did learn the sequence throughout the experiment, but did not need to recall it in the cued blocks, because the cue was giving all necessary spatial information. In the uncued blocks, participants were able to recall the sequence even without the assistance of directional given cues. This was not the case for the SOC-HV condition. The results of the OBJ-HV condition are in line with the study by Cleeremans (1997). In this study, participants also performed in a dual-stimulus SRT task in a dual-stimulus experiment with additional directional cues. Results show that participants seem to have learnt the hidden sequence throughout the training blocks in the HV condition, but only express this knowledge when exposed to the trials without directional cues. However, this is only true for the OBJ-HV condition in our experiment and not for the SOC-HV condition. In addition, descriptively, it seems that the participants were able to recall the training sequence, however, this finding was not significant.

Looking at the LV conditions, both cue types elicit a SLS in the uncued blocks, even no SLS was measured for the cued blocks. This finding suggests that participants were indeed able to learn the hidden sequence but could not use this knowing when a distracting cue was present. This could mean that this implicit learning can only be assessed when no other cognitive tasks were active, like trying to ignore the cue. As soon as no distracting cueing is present, participants are able to recall the learnt sequence.

If asked explicitly, as in the inclusion generation task for all combinations of cues and cueing validity, participants were not able to recall the sequence, independent of the given combination of cue and cueing validity, even in those conditions, in which participants had a learning score in the uncued blocks.

In the exclusion generation task, both cues, i.e., the social and object cue, elicit a positive score, meaning that participants were not able to suppress the implicitly learnt sequence and thus they were recreating parts of the learnt sequence. These results suggest that no combination of cue type and cueing validity facilitate implicit learning.

Performance in both exclusion and inclusion task conditions was impaired under dual-stimulus conditions, which suggest that the influence of validity and cue is not specific to any type of knowledge. Instead, it results in a general overload of the attentional system, despite the fact participants showed a learning effect in the cued blocks in the SOC-HV condition.

Interestingly, in two SRT studies without cueing, individual strategies of processing implicit sequences could be demonstrated in all those cases in which the implicit sequences became explicit, based on behavioral responses (Yordanova et al., 2015) as well as neuronal activity (Verleger et al., 2015). It is conceivable that the individual processing strategies might also be influenced by the presence of additional directional cues, as used in our study. Unfortunately, we are not able to investigate these underlying processes in the present study due to the small amount of persons with explicit knowledge of the hidden sequence.

## Conclusion

This study investigated the influence of two cue types, i.e., social and non-social/object, with different cueing validities, high and LV, on motor responses. In addition, the study investigates the influence of the combination of stimulus and cueing validity on implicit motor learning.

Our results highlight the finding that a gaze-mediated social context significantly affects mechanisms of action processes, but only under circumstances of uncertainty, suggesting that the processing of inner mental motivations of another person are interfering with the implicit motor learning task employed in this study as experimental vehicle.

These findings are consistent with the idea of a modulatory effect of gaze-mediated social contexts on action control, leading to faster responses when interacting with a social cue in contrast to an object cue. Interestingly and consistent with the idea of mentalizing being initiated and entertained in the presence of a “person”, high valid directional cues given by an object stimulus were perceived as more robust and, hence, more helpful than social cues.

In addition, we show that implicit motor learning is—albeit its automatic process nature—dependent on the environment of the task.

## Data Availability

The raw data supporting the conclusions of this manuscript will be made available by the authors, without undue reservation, to any qualified researcher.

## Author Contributions

AG, AC, GB and KV designed the experiment. AG collected and analyzed the data and wrote the manuscript. AG, AC and KV were involved in the interpretation of the results. AC, GB and KV were involved in the revision of the manuscript.

## Conflict of Interest Statement

The authors declare that the research was conducted in the absence of any commercial or financial relationships that could be construed as a potential conflict of interest.

## Footnotes

^1^www.neurobs.com
